# Supplementary figures and images for: Extracellular vesicles derived from DFO-preconditioned canine AT-MSCs reprogram macrophages into M2 phase
Source: PLoS One. 2021 Jul 26;16(7):e0254657. doi: 10.1371/journal.pone.0254657 (PMC8312919; doi:10.1371/journal.pone.0254657)

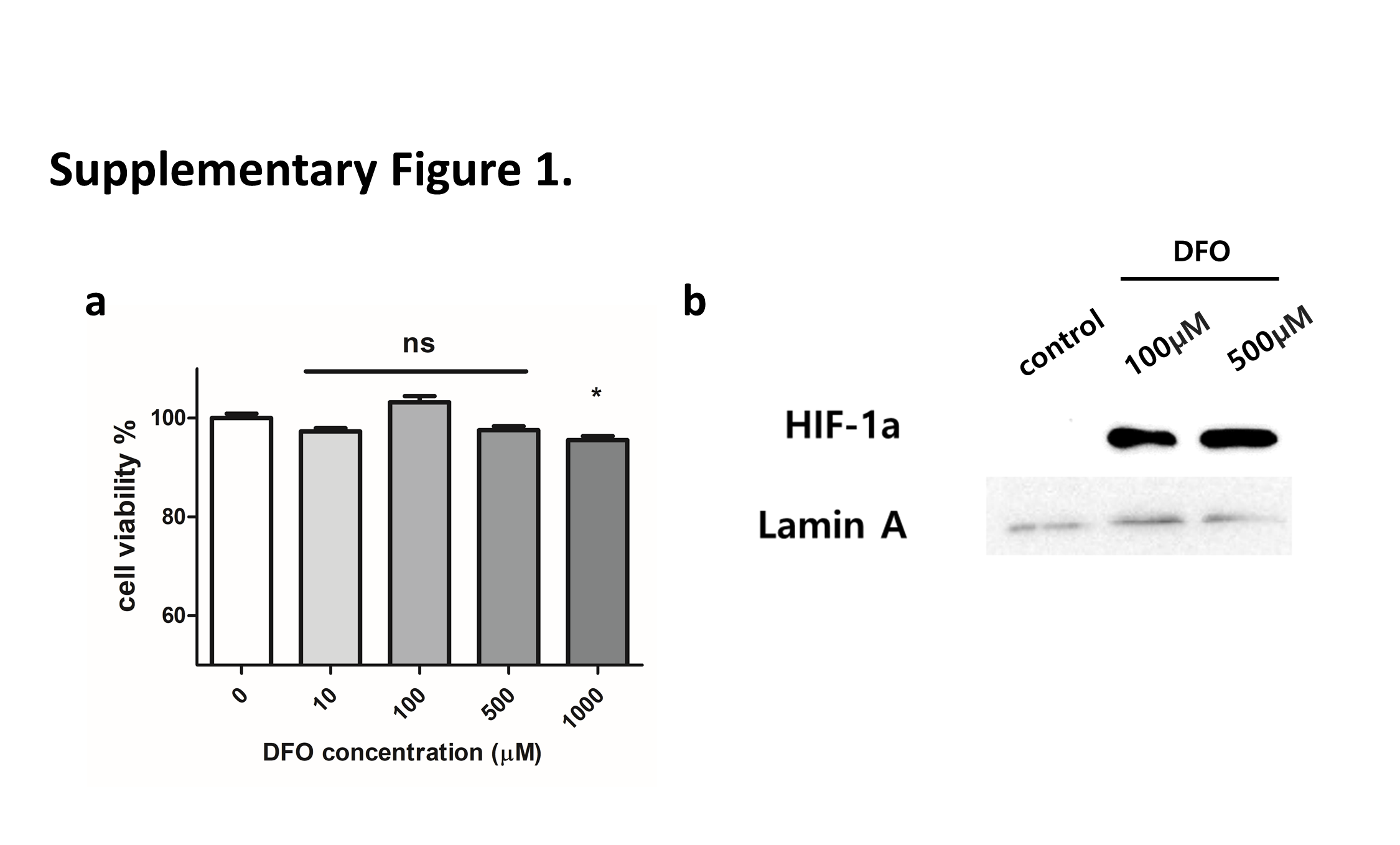

Supplement: S1 Fig — (a) Depending on DFO concentration, cell viability was not affected under 500 μΜ and was decreased in 1mM. (b) HIF-1α was accumulated in the nuclear of cAT-MSCDFO, which showed that DFO treatment could accumulate HIF-1α in cAT-MSC nucleus. Results are shown as means ± standard deviation. *P < 0.05. ns, not significant. (TIF) [file pone.0254657.s001.TIF]
