# Supplementary material for: Extracellular vesicles derived from DFO-preconditioned canine AT-MSCs reprogram macrophages into M2 phase
Source: PLoS One. 2021 Jul 26;16(7):e0254657. doi: 10.1371/journal.pone.0254657 (PMC8312919; doi:10.1371/journal.pone.0254657)

Figure 1. j

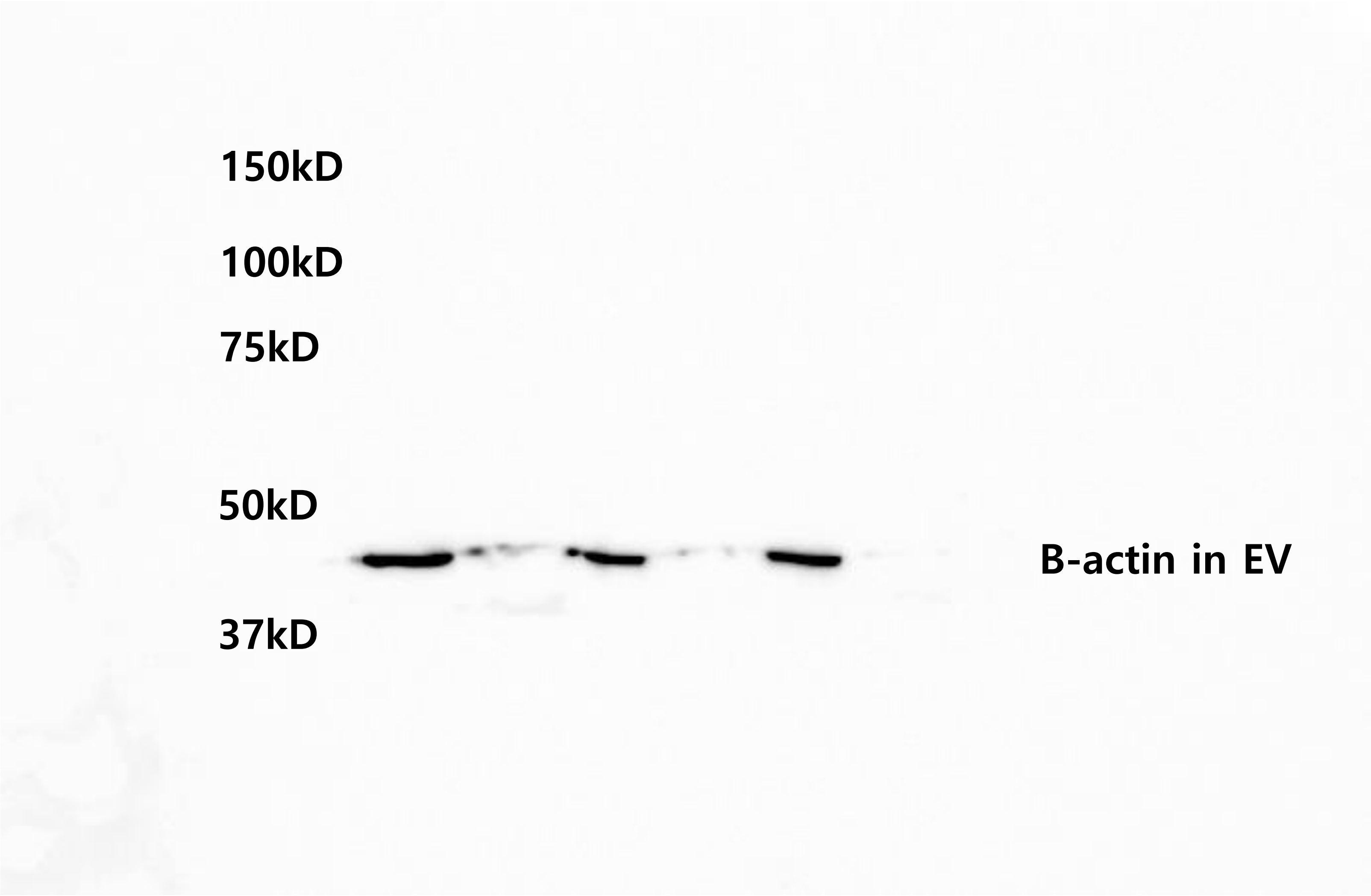

Figure 1. j

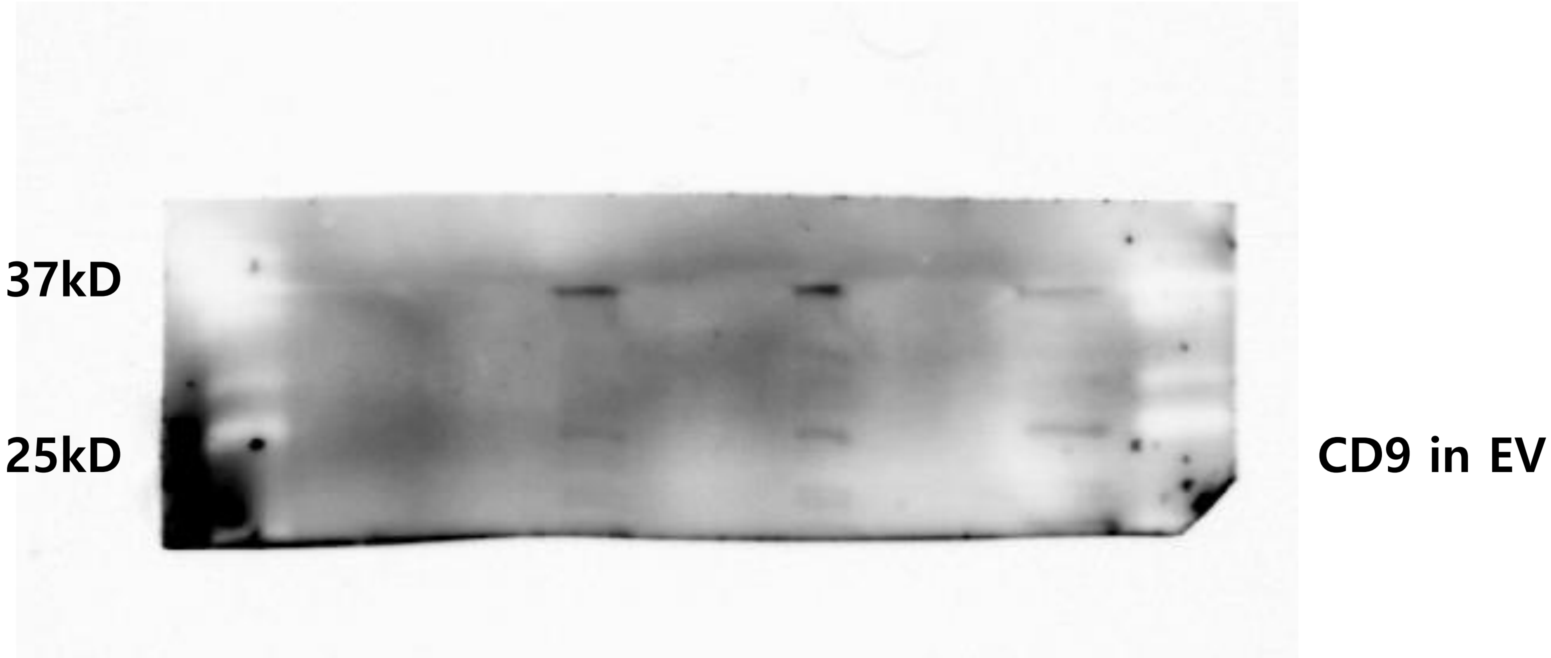

Figure 1. j

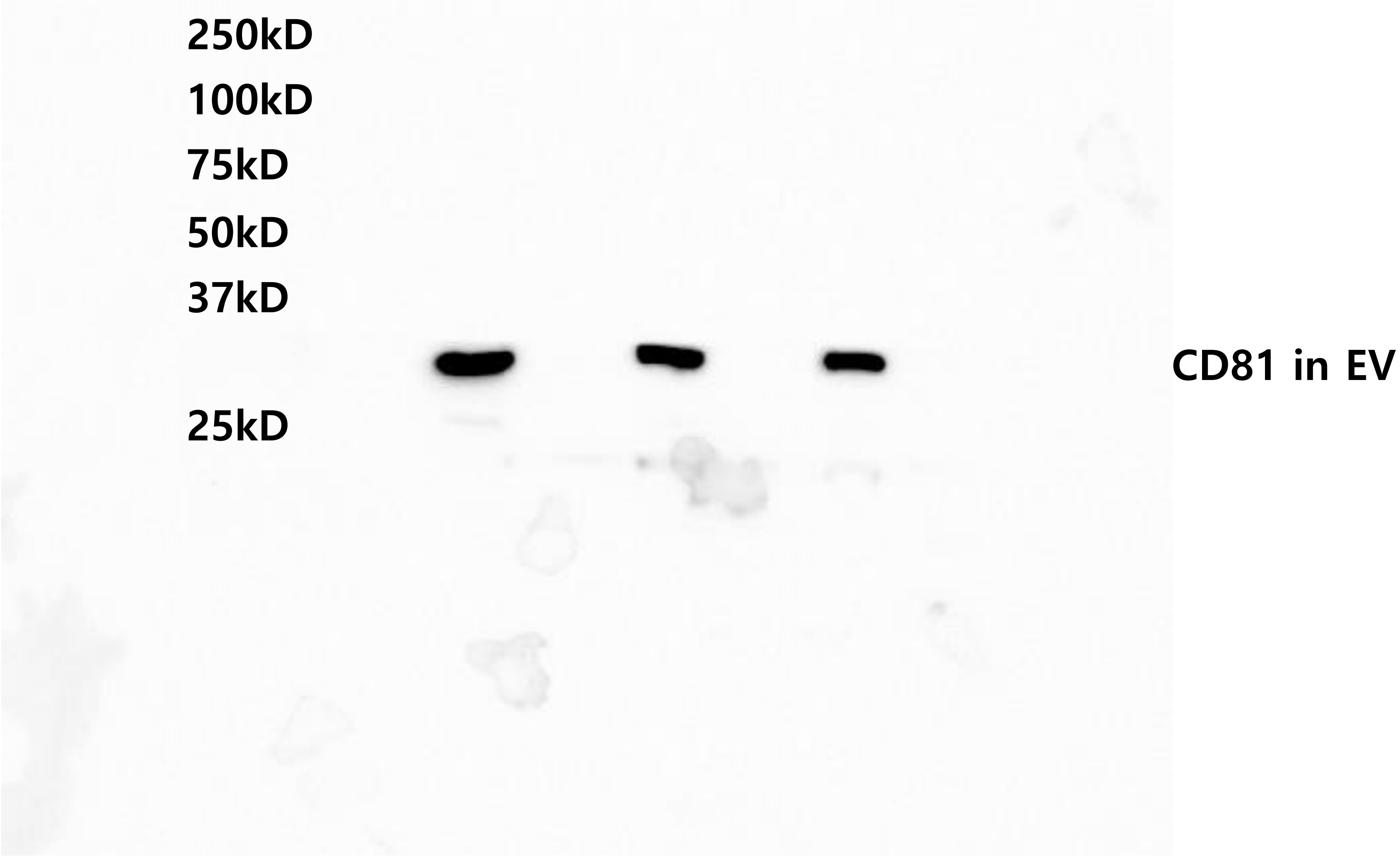

Figure 2. b

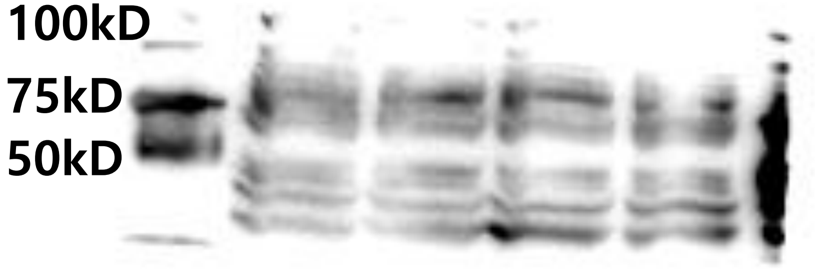

COX-2 in MSC

Figure 2. b

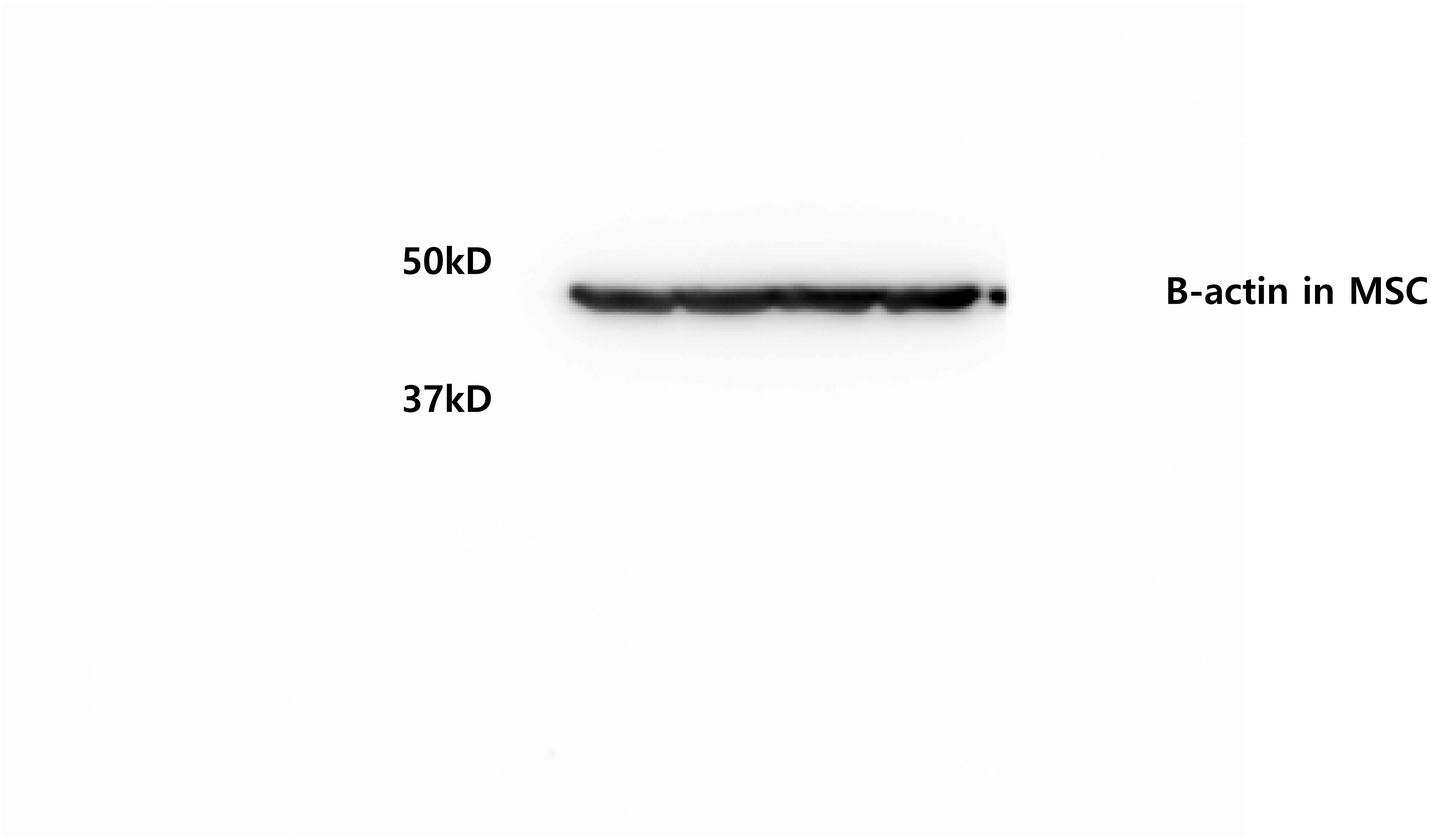

Figure 3. a

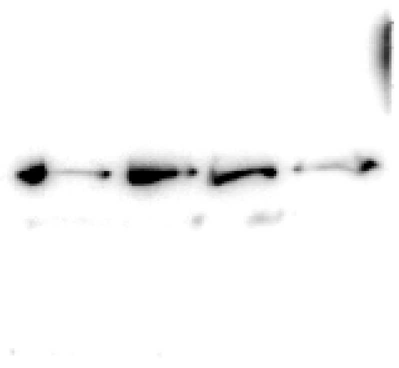

Figure 3. b

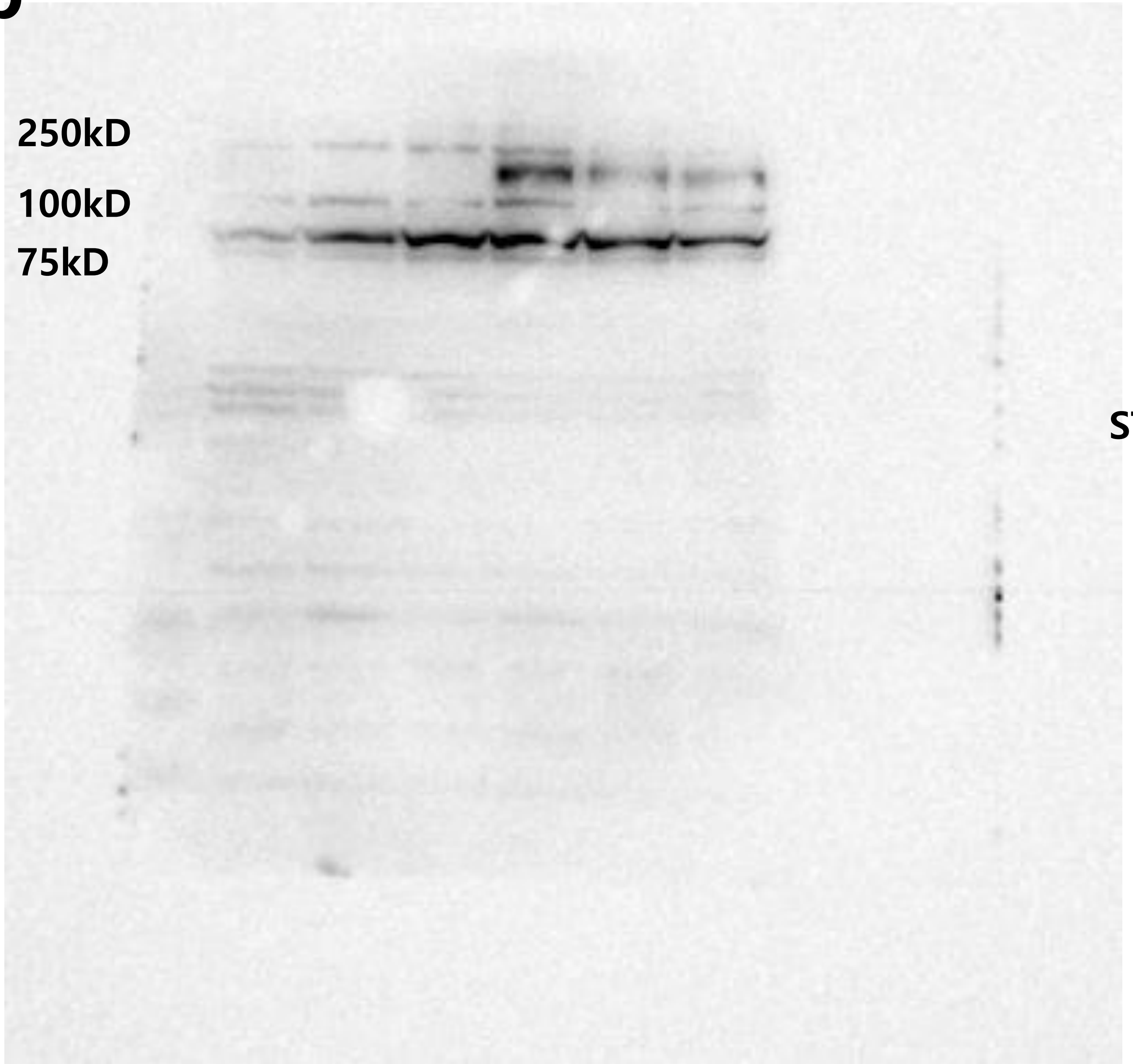

STAT3 in DH82

Figure 3. b

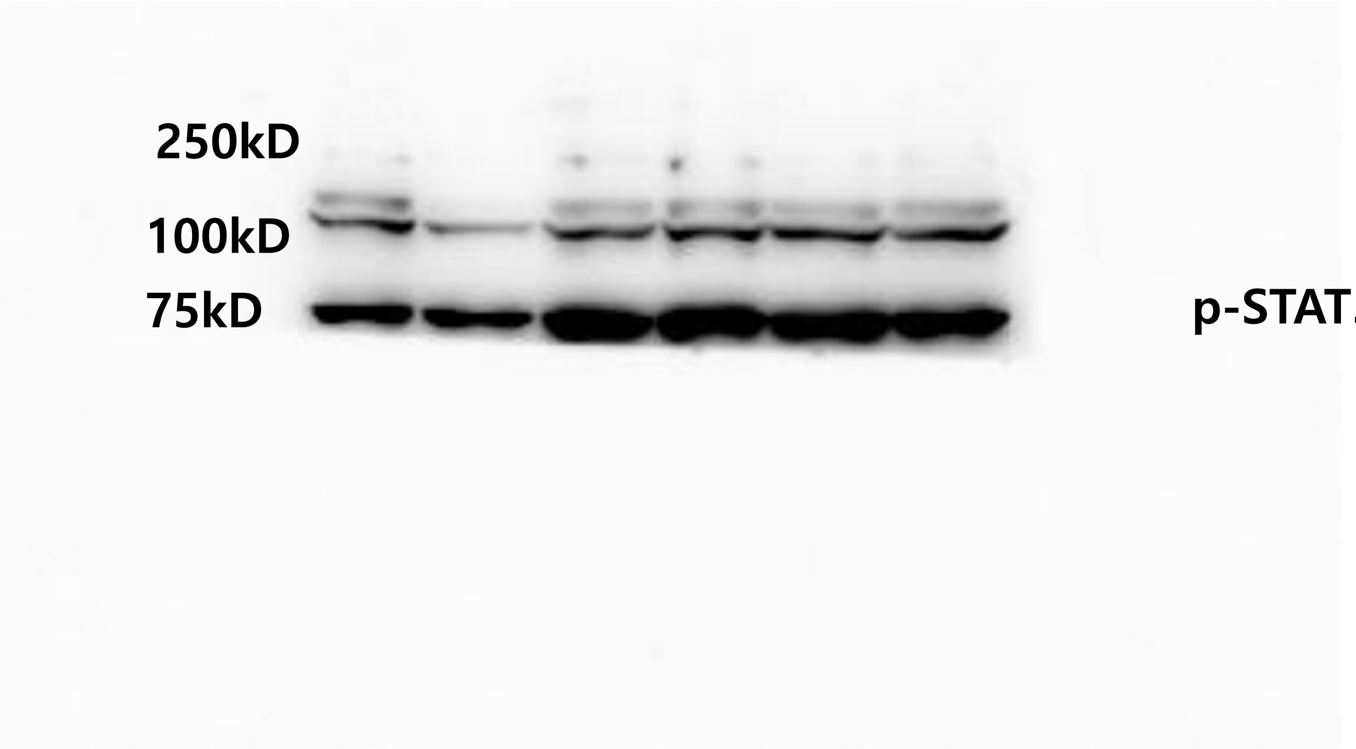

p-STAT3 in DH82

Figure 3. b

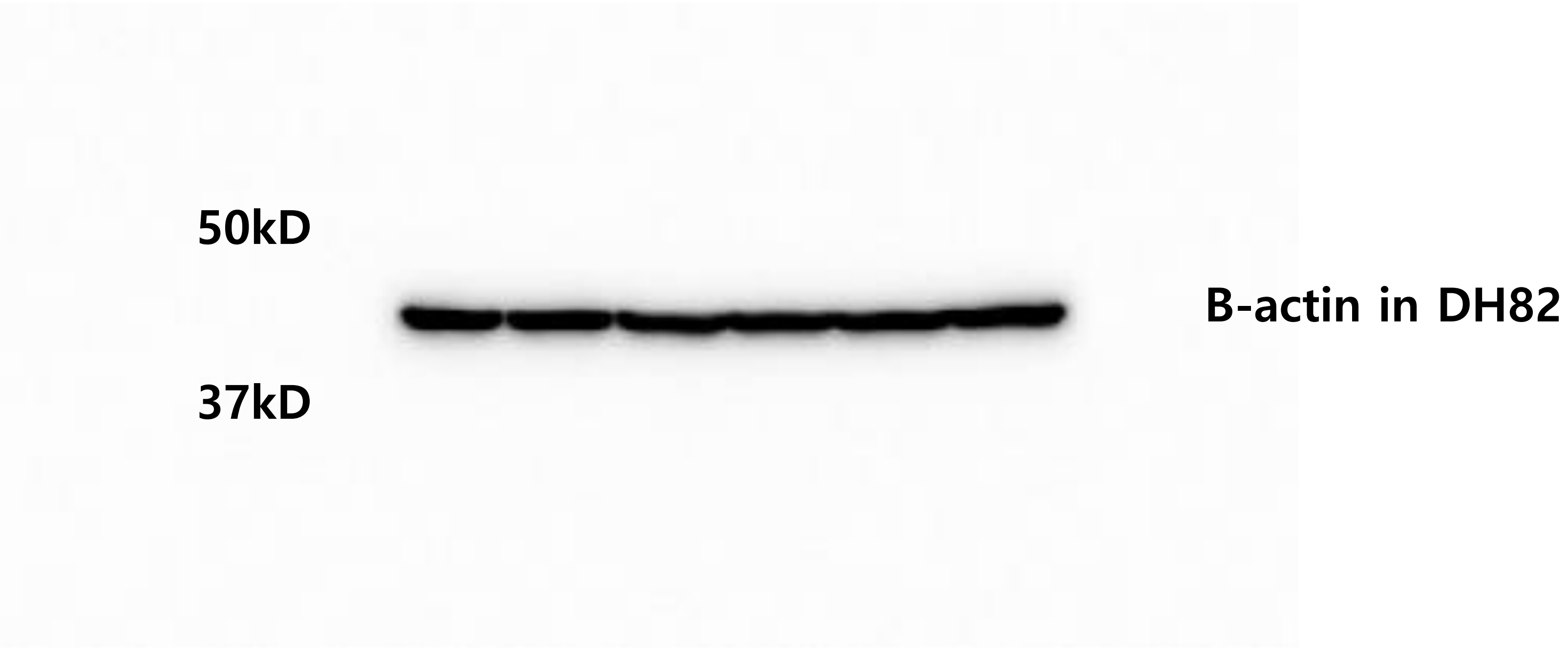

Supplement Figure 1. b

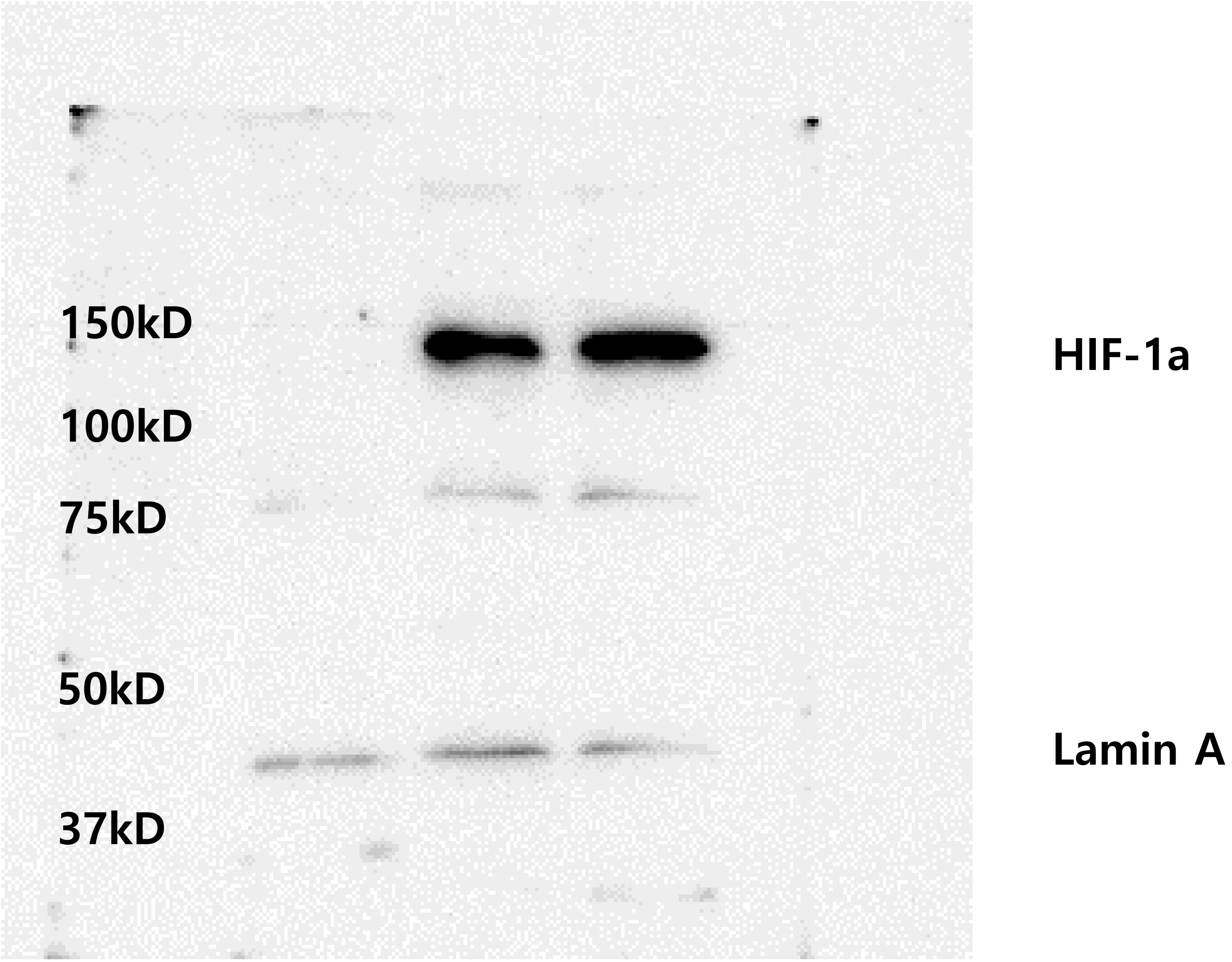

Supplement: S1 Raw images — (PDF) [file pone.0254657.s002.pdf]
